# Lung Imaging in Acute Hypoxemic Respiratory Failure: From Physics to Bedside Applications

**DOI:** 10.3390/jcm15114345

**Published:** 2026-06-04

**Authors:** Silvia Coppola, Tommaso Pozzi, Davide Chiumello

**Affiliations:** 1 Department of Health Sciences, University of Milan, 20142 Milan, Italy; davide.chiumello@unimi.it; 2 Department of Anesthesia and Intensive Care, ASST Santi Paolo e Carlo, San Paolo University Hospital Milan, 20142 Milan, Italy; tommaso.pozzi94@gmail.com; 3 Coordinated Center on Respiratory Failure, University of Milan, 20157 Milan, Italy

**Keywords:** acute hypoxemic respiratory failure, lung imaging, chest X-ray, computed tomography, lung ultrasound, electrical impedance tomography, positron emission tomography

## Abstract

Acute hypoxemic respiratory failure (AHRF) represents one of the most common and clinically challenging indications for invasive mechanical ventilation in the intensive care unit, characterized by profound etiological heterogeneity that demands accurate diagnosis to guide treatment. While clinical history, physical examination, and laboratory data remain essential, they are often insufficient to reliably discriminate among conditions such as acute respiratory distress syndrome (ARDS), cardiogenic pulmonary edema, and pneumonia—particularly in mechanically ventilated patients. Lung imaging has therefore emerged as an indispensable complement to clinical assessment. In this narrative review, we systematically describe the physical principles, clinical applications, and limitations of the imaging modalities currently available in critical care: chest X-ray (CXR), computed tomography (CT), lung ultrasound (LUS), electrical impedance tomography (EIT), and positron emission tomography (PET). CXR remains the most widely used bedside tool but is constrained by low sensitivity and significant interobserver variability. CT is the gold standard for morphological and quantitative lung phenotyping, enabling the assessment of recruitability, baby lung characterization, and the identification of complications, but requires patient transport and exposes patients to ionizing radiation. LUS offers real-time, bedside evaluation of aeration with high diagnostic accuracy for pneumothorax and pleural effusion, and is increasingly integrated into revised ARDS diagnostic criteria. EIT enables continuous, radiation-free monitoring of regional ventilation distribution and positive end-expiratory pressure (PEEP)-guided titration directly at the bedside. While PET provides unparalleled quantification of regional inflammation and ventilation-perfusion mismatch, it currently remains a purely investigative research tool. Finally, we discuss emerging technological and AI-driven advances—including dual-energy CT, next-generation EIT, and deep learning algorithms—that are poised to transform lung imaging from a passive diagnostic tool into an active, personalized guide to respiratory management.

## 1. Introduction

Acute hypoxemic respiratory failure remains one of the leading indications for invasive mechanical ventilation, carrying substantial morbidity and mortality in intensive care units [1]. The clinical challenge posed by acute hypoxemic respiratory failure (AHRF) lies not only in its severity, but also in its profound etiological heterogeneity. Conditions as diverse as acute cardiogenic pulmonary edema, pneumonia, acute exacerbations of chronic obstructive pulmonary disease, and acute respiratory distress syndrome (ARDS) may present with overlapping clinical features, yet demand markedly different therapeutic strategies [2]. Accurate etiological diagnosis is therefore essential to guide treatment and inform prognosis [3]; historically, it has relied on the integration of clinical history, physical examination, arterial blood gas analysis, and laboratory markers. However, this approach is often insufficient to reliably discriminate among these entities—particularly in mechanically ventilated patients, in whom bedside examination is inherently constrained and the clinical picture is continuously reshaped by ongoing interventions [2]. In this context, lung imaging has emerged as an indispensable complement to clinical assessment, enabling morphological and quantitative characterization of the lung, chest wall, and airways through direct visualization. A broad spectrum of modalities is now available in clinical practice, each illuminating different facets of respiratory pathophysiology.

**Figure 1:**
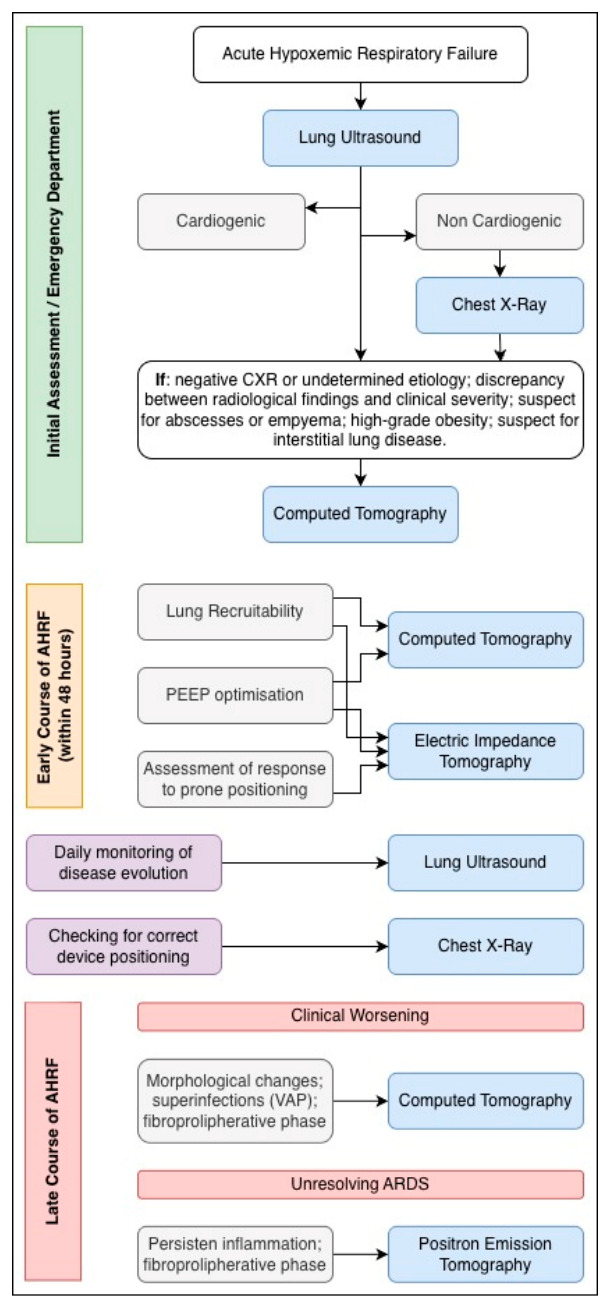
Diagnostic imaging work-up for acute hypoxemic respiratory failure.

In this narrative review, we summarize the currently available lung imaging techniques, outlining their clinical indications, applications, and limitations in the management of acute hypoxemic respiratory failure (Table 1), proposing a diagnostic imaging work-up (Figure 1). Additionally, we discuss recent technological advances and the emerging role of artificial intelligence (AI) in improving lung imaging.

## 2. Chest X-Ray

### 2.1. Physics and Overview of the Modality

Chest X-ray (CXR) imaging relies on the attenuation of photon beams, generated by an X-ray tube, as they traverse the thorax. According to the Beer–Lambert law, attenuation depends on tissue density and atomic number: high-density structures such as bone or consolidated lung appear radiopaque (white), air-filled regions appear radiolucent (dark), and soft tissues produce intermediate shades of gray. A detector, positioned opposite the tube, converts the transmitted photons into a digital two-dimensional projection image [4,5]. In intensive care units (ICUs), antero-posterior projections acquired in the semi-upright or supine position are standard practice, offering a comprehensive view of the lung parenchyma and thoracic extrapulmonary structures; lateral views are rarely feasible in critically ill patients and add limited information on pleural effusion extent [6]. The average effective dose per a single antero-posterior examination is approximately 0.01–0.02 mSv. Modern systems are digital, with portable units enabling bedside imaging in the critically ill [7].

### 2.2. Clinical Applications

**Figure 2:**
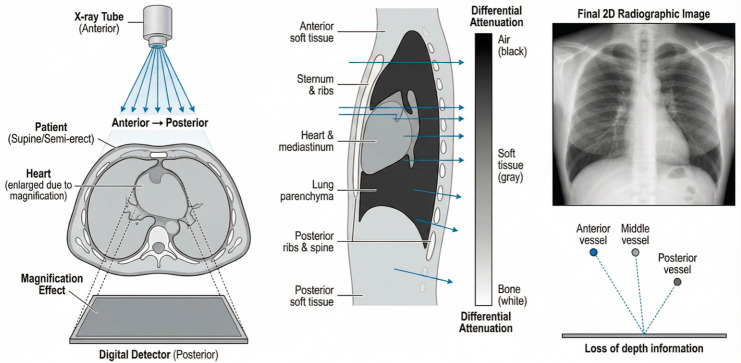
Instrumental setting (**Left**), attenuation scale (**Center**), and final bidimensional rendering (**Right**) for chest X-ray (CXR) for lung imaging. (**Left**): the anterior–posterior setting results in a bidimensional projection with a magnification effect due to the geometry of patient positioning. (**Center**): different tissues result in different attenuation patterns (i.e., air is perceived as black, while dense tissues are seen as white). (**Right**): the final radiographic image from CXR suffers from the magnification effect and the overlapping of multiple structures caused by the flattening of a tridimensional structure into a bidimensional image.

A CXR is the most frequently prescribed technique for the initial evaluation of patients with AHRF. In this context, indications for antero-posterior CXR include acute chest pain, acute respiratory symptoms (e.g., dyspnea, respiratory distress, hypoxia, cough), critical illness with abnormal vital signs, trauma, and unexplained fever; or it can be used after the placement of central venous lines, endotracheal tubes, and chest tubes to check the correct position of these devices [6]. Thanks to their portability, rapidity, repeatability, and the fact that they can be performed at the bedside without the need to transport critically ill patients, CXRs are currently the most widely used method for re-evaluating the lung parenchyma in ICUs. While there is no consensus on how often CXR should be performed during an ICU stay, two meta-analyses have shown that abandoning daily routine CXR re-evaluation does not lead to negative outcomes in terms of either hospital or ICU mortality or length of stay [8,9]. Indeed, CXR re-evaluation is recommended for patients when exhibiting clinical changes indicating a deterioration in respiratory or cardiac function (Figure 2) [9].

### 2.3. Limitations

Although portable, rapid, and associated with low radiation doses, CXR suffers from limited sensitivity, modest specificity across different parenchymal pathologies, and substantial inter-observer variability. A recent systematic review and meta-analysis reported an overall sensitivity of only 40–58% for detecting lung pathology in critically ill patients with respiratory symptoms, using chest computed tomography (CT) as the reference standard; accuracy was particularly poor for pneumothorax and lung contusion, and comparatively better for interstitial syndrome, pleural effusion, and consolidation. Lung ultrasound consistently outperformed CXR in sensitivity across all these conditions, while specificity remained high for both modalities [10]. A subsequent study further showed that interobserver agreement in CXR interpretation is only fair [11]. Taken together, these limitations support a more selective use of CXR in ICU, primarily for verifying device placement or evaluating abrupt clinical deterioration [10].

## 3. Computed Tomography

### 3.1. Physics and Overview of the Modality

The physical principle behind CT scans is identical to that of CXR, and is based on the emission of photons from an X-ray tube. While in CXR, the radiogenic tube is fixed and directed unidirectionally towards the patient’s chest; during CT imaging a complex sensor array detects the attenuation of the X-ray beam while rotating along with the beam generator, with an accuracy thousands of times greater than that of a CXR: this helical path allows for the representation of each spatial unit in the explored field (i.e., voxel) according to the density of the tissue, which is then reconstructed with an arbitrary grayscale from black (i.e., air) to white (i.e., bone). This implies a higher electrical tension in the radiogenic tube (e.g., 80–140 kV) and, thus, a higher radiation exposure (Table 2) [12,13].

### 3.2. Clinical Applications

#### 3.2.1. Etiology and Complications

Due to the possibility to image the lung in three dimensions with high accuracy and reduced execution time, CT represents the gold standard imaging technique to investigate radiological lung characteristics during AHRF [3,6,14]. Indeed, although not formally included in the Berlin ARDS definition or its expanded modifications [15,16], CT excels at identifying the hallmark radiological characteristics of ARDS (i.e., consolidation in the dependent lung regions and diffuse ground-glass opacities reflecting alveolo-capillary syndrome). It is useful in distinguishing ARDS from its mimics, such as diffuse alveolar hemorrhage and chronic interstitial lung disease. Moreover, CT reliably detects complications that are frequently not diagnosed by CXR, such as pneumothorax, pneumomediastinum, and pulmonary embolism [14]. Recently, CT scan enabled the distinctive features of COVID-19-associated respiratory failure, characterizing the early course of the disease not as a predominant alveolar disease, which lacks the hallmark features of ARDS, and pointing towards ventilation–perfusion mismatch to explain the degree of hypoxemia encountered [17].

#### 3.2.2. Morphological Analysis

Thanks to its spatial resolution, lung CT allows for the morphological phenotyping of AHRF in distinct patterns (i.e., focal, with consolidations mostly in dependent lung regions, or diffuse, with contiguous areas of poorly and well aerated lung tissue across the entire lung). Constantin et al. demonstrated how administering ventilatory strategies not aligned with morphological phenotypes led to an increase in mortality rates in ARDS patients [18]. Indeed, while focal ARDS harbors substantial volumes of already-aerated, non-dependent lung tissue vulnerable to overdistension with higher positive end-expiratory pressure (PEEP) levels and recruitment maneuvers, diffuse ARDS generally has a more homogeneously recruitable lung and may benefit from higher PEEP [18].

#### 3.2.3. Quantitative Analysis and Baby Lung Characterization

By measuring regional tissue density in three dimensions, CT has demonstrated the heterogeneity of lung inflation in supine ARDS patients, which exhibit a vertical gradient of aeration: pulmonary hyperdensities predominate in the dorsal (i.e., dependent) lung, while the ventral (i.e., non-dependent) lung regions remain relatively aerated [19]. In the 1980s, CT studies paved the way for the identification of the baby lung as an anatomical unit consisting of parts of the lungs with preserved aeration mainly located in the non-dependent lung [20], while dependent lung regions suffer from a high superimposed pressure due to lung edema, which promotes atelectasis [21]. The paradigm shift from the idea of a globally hardened lung to the radiological evidence of a functionally and anatomically smaller lung, but with preserved mechanical properties, was the basis for the subsequent development of protective ventilation protocols during ARDS [22].

#### 3.2.4. Lung Recruitability

Offline analysis of lungs from CT scans enables precise quantification of the aeration of each voxel by examining its density and expressing it as an attenuation coefficient in Hounsfield units (HU). Once voxel densities are measured, lung parenchyma is partitioned into four distinct compartments based on reference Hounsfield thresholds: non-aerated, poorly aerated, normally aerated, and hyperinflated lung tissues [23]. Measuring the extent of each aeration compartment at two different airway pressures has allowed for the characterization of lung recruitment, which indicates the amount of collapsed lung units (i.e., the difference in grams of non-aerated tissue, according to Gattinoni et al. [23] or non-aerated volume according to Borges et al. [24]) which became viable for aeration at increased airway pressure. The level of airway pressure causing lung opening and closing has been a matter of debate: despite the fact that they both used two lung CT scans, Gattinoni et al. [23] proposed evaluating lung recruitability at 5 and 45 cmH_2_O, while Borges et al. advocated for administering pressures up to 60 cmH_2_O [25]. The amount of lung recruitability according to these two methods has been demonstrated to be a marker of the disease severity and to be associated with mortality in ARDS [23]. Conversely, Malbouisson et al. proposed another method to quantify lung recruitability, based on the amount of gas penetrating into non-aerated or poorly aerated compartments, which correlated with the improvement in arterial oxygenation with PEEP [26].

#### 3.2.5. Prone Positioning

**Figure 3:**
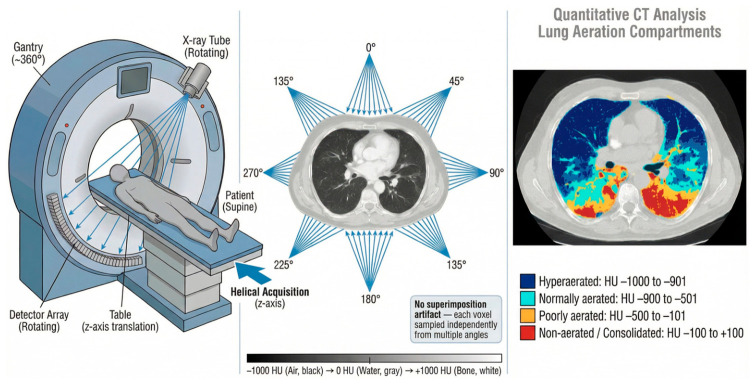
Instrumental setting (**Left**), acquisition angles with attenuation scale (**Center**), and quantitative lung analysis according to lung aeration (**Right**) for Computed Tomography (CT). (**Left** and **Center**): the rotating X-ray tube allows for the investigation of the thoracic structures from multiple angles, without any spatial distortion during reconstruction; (**Center** and **Right**): similarly to CXR, signal attenuation is proportional to aeration, and it is visualized as black–white scale. (**Right**): a typical basal CT slice from a patient with acute respiratory distress syndrome, highlighting the distribution of hyperaerated (blue), normally aerated (light blue), poorly aerated (yellow), and non-aerated (red) lung compartments, as detected with quantitative lung CT analysis.

By imaging the lungs in the supine and prone positions after one another, in the early 1990s, Gattinoni et al. demonstrated the redistribution of lung densities from dorsal to ventral lung regions [27]. Subsequent CT-based studies have demonstrated that prone positioning reduces the mass of non-aerated and poorly aerated lung tissue in non-dependent lung regions [28], thus leading to a more homogeneous distribution of transpulmonary pressures across the ventral-to-dorsal axis [29]. Experimental studies have also used CT to demonstrate that prone positioning leads to an improved ventilation–perfusion matching [30] (Figure 3).

### 3.3. Limitations

The routine use of CT scans for serial monitoring in patients with AHRF is limited by the need to transport critically ill patients (often requiring invasive ventilated and being hemodynamically unstable) to the radiology suite. This process could be complicated by inadvertent extubation and ventilatory disconnection, as well as hemodynamic decompensation [31]. There is also concern about the cumulative exposure to ionizing radiation.

## 4. Lung Ultrasound

### 4.1. Physics and Overview of the Modality

Because ultrasound cannot penetrate an aerated lung, lung ultrasound (LUS) relies on the interpretation of the pleural line and the artifacts generated at this interface. Image formation depends on two principles: the travel time of the ultrasound beam between transducer and reflecting interface, which encodes depth, and the intensity of reflection—stronger reflections appear white, weaker reflections gray, and non-reflective aerated regions black [14]. Transducer selection should match the clinical question: high-frequency linear probes (~10 MHz) offer superior resolution of superficial structures such as the pleural line but limited penetration, whereas low-frequency convex or microconvex probes (1–5 MHz) provide greater depth of field at the expense of surface resolution [32].

Lung ultrasound interpretation is therefore artifact-based, with patterns reflecting the degree of tissue aeration [33]. The normally aerated lung is characterized by A-lines—regularly spaced hyperechoic horizontal reverberations of the pleural line—accompanied by respiratory lung sliding or transmitted cardiac motion (lung pulse). Interstitial syndrome, reflecting loss of aeration from interstitial or alveolar edema or atelectasis, manifests as B-lines: hyperechoic, laser-like vertical artifacts arising from the pleural line, extending to the edge of the screen without fading, and moving with tidal breathing. The number of B-lines is inversely related to aeration: normally aerated areas show A-lines or fewer than three B-lines per intercostal space, moderate loss of aeration corresponds to more than three scattered B-lines, and severe loss to more than three coalescent B-lines. Near-complete loss of aeration—lung consolidation—appears as a tissue-like, hypoechoic image arising from the pleural line [33].

### 4.2. Clinical Applications

LUS typically reveals a heterogeneous interstitial syndrome with asymmetric B-lines, patchy consolidations, and interposed areas of preserved aeration, a pattern that aids the identification of focal versus non-focal ARDS subphenotypes [34,35]; accordingly, LUS has been incorporated as a diagnostic tool in the revised ARDS definitions, including the Kigali modification of the Berlin criteria [16]. In mechanically ventilated patients, LUS enables daily monitoring of lung re-aeration in response to clinical interventions such as antibiotic therapy, recruitment maneuvers, and prone positioning [33], and has been used to guide PEEP titration by tracking the disappearance of B-lines in collapsed regions [36,37]. Wang et al. [38] further showed that LUS can predict re-aeration of dorsal segments in response to prone positioning [39,40,41]. Whole-lung LUS scoring has proven more accurate than visual assessment of consolidated areas for quantifying alveolar–interstitial syndrome and re-aeration. The semiquantitative score proposed by Bouhemad et al.—assigning 0 points for normal aeration, 1 for moderate loss (B1), 2 for severe loss (B2), and 3 for consolidation—has become the standard quantitative tool in LUS practice [42].

The LUS pattern of acute heart failure is characterized by multiple B-lines in all thoracic regions, which is typical of a diffuse bilateral interstitial syndrome. As with ARDS, the number of B-lines in the LUS pattern correlates with the amount of lost aeration and extravascular lung water [43]. However, unlike the patchy distribution of ARDS, cardiogenic edema shows a more homogeneous, gravity-dependent pattern of B-lines prevalence. In acute heart failure, the number of B-lines has typically been found to correlate not only with other markers of pulmonary edema [44,45,46], but also with the response to diuretic therapy or hemodialysis [47].

LUS can accurately diagnose pneumothorax with 98% specificity and 78% sensitivity [48], which is better than CXR [49,50]. In this case, the LUS pattern may comprise A-lines and the absence of lung sliding and B-lines, as well as the visualization of a lung point, corresponding to the area of the chest wall adjacent to the pneumothorax where respiratory lung movement reappears. The border between the sliding and non-sliding patterns provides an indication of the extent and volume of the pneumothorax. Notably, the sole presence of absent lung sliding does not guarantee a 100% specificity for pneumothorax, as it can be present in other clinical conditions [51].

Similarly, the diagnostic accuracy of LUS for pleural effusions is very high, with a specificity and sensitivity of 94–98% [52]. LUS enables the semiquantitative measurement of the fluid volume, reducing the risk of pneumothorax to <1% during thoracentesis in mechanically ventilated patients [53].

**Figure 4:**
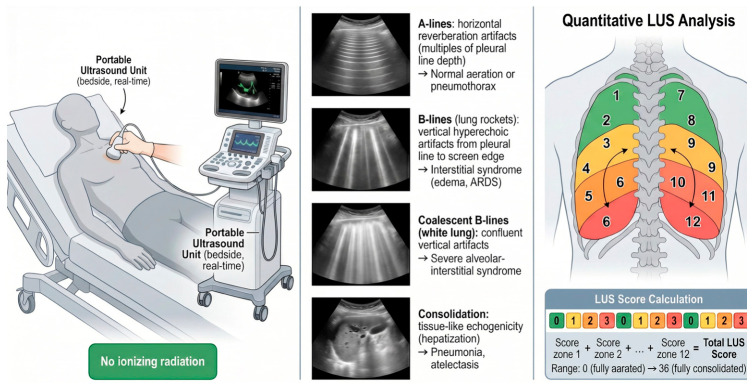
Instrumental setting (**Left**), artifact classification (**Center**), and quantitative aeration score (**Right**) for Lung Ultrasound (LUS). (**Left**): instrumental setting and patient positioning during LUS examination; (**Center**): LUS images according to progressive loss of aeration from the top (A-lines) to the bottom (Consolidation). (**Right**): distribution of lung segment for quantitative analysis of aeration during LUS.

In both spontaneous breathing and mechanically ventilated patients, LUS is a valid tool for bedside diagnosis of lung consolidation and allows for the daily monitoring of changes in aeration in patients treated with antibiotics [10,54]. Mongodi et al. compared the Clinical Pulmonary Infection Score (CPIS) to an LUS-based score for predicting ventilator-associated pneumonia and found that the LUS score, based on the presence of subpleural and lobar consolidations and dynamic arborescent and linear air bronchograms, had a higher sensitivity and specificity [54]. Lichteinstein et al. demonstrated that the findings of a static air bronchogram (i.e., which shows no translational movement with respiration) were associated with the presence of atelectasis, whereas a dynamic air bronchogram (i.e., hyperechoic foci which clearly move in synchrony with tidal breathing) indicated the presence of pneumonia [55] (Figure 4).

### 4.3. Limitations

Although 25 supervised lung ultrasound examinations have been demonstrated to represent an adequate number to achieve minimal competence for LUS, some automated algorithm has been found with conflicting results in terms of clinical accuracy in detecting LUS pattern. A major limitation, however, is the inability to distinguish true alveolar recruitment from mere inflation: LUS is insensitive to hyperinflation, which may lead to inappropriate PEEP escalation and hemodynamic compromise [3].

## 5. Electrical Impedance Tomography

### 5.1. Physics and Overview of the Modality

Electrical impedance tomography (EIT) is a non-invasive bedside technique that reconstructs tomographic images of the lungs from variations in tissue electrical impedance. A belt embedded with 16 or 32 electrodes is placed around the thorax at the fourth–fifth intercostal space; pairs of electrodes sequentially inject a low alternating current (5–10 mA) while the remaining electrodes record the resulting voltages, with the excitation pattern rotating around the chest up to 50 times per second. Because air is a poor conductor, thoracic impedance rises during inspiration and falls during expiration, allowing for the reconstruction of a two-dimensional map of regional aeration [14].

EIT quantifies only relative changes in global and regional air content with respect to a baseline set at the start of acquisition. Two main parameters are derived: end-expiratory lung impedance (EELI), which tracks changes in functional residual capacity, and tidal impedance variation (TIV), the end-inspiratory to end-expiratory impedance difference, which reflects tidal volume distribution on a breath-by-breath basis. Impedance changes, however, are not specific to air content: accumulation of lung fluid (e.g., edema or fluid loading) and alterations in tissue conductivity (e.g., cell rupture) can produce comparable signals [56,57].

Changes in end-expiratory lung impedance are proportional to variations in end-expiratory lung volume, which may be caused by ventilatory settings adjustment or the progression of lung disease itself. Differently, tidal impedance variations for the whole lung and for different regions are proportional to the distribution of air volume in the whole lung or in each region. End-expiratory and tidal impedance variations can be calculated for the whole lung or in pre-specified lung regions (i.e., regions of interests, or ROIs), which can be set as quadrants to monitor ventilation distribution at a regional level. Therefore, EIT can be used to assess both global and regional volume distribution according to changes in pulmonary mechanical properties or ventilatory settings. By comparing global and pixel-level impedance changes, EIT can provide the global inhomogeneity index to quantify ventilation distribution heterogeneity. Finally, monitoring regional aeration distribution allows for the computing of a regional compliance map derived from the ratio between regional tidal impedance variation to driving pressure [58].

Although it still has a limited clinical applicability, EIT has been used to evaluate lung perfusion distribution. This can be achieved by injecting a hypertonic saline solution, which temporarily alters blood impedance by changing its osmolality. Alternatively, pulsatile cardiac signal analysis can be used, based on post hoc algorithm analysis. These changes can be transformed by EIT to provide a regional perfusion map [59,60].

Many conditions may affect EIT data reliability and interpretation, both referring to the patient (e.g., extreme obesity, which is sometimes considered as a relative contraindication, unstable chest trauma or wounds, extreme diaphoresis, agitation) or to the operator (e.g., correct belt positioning) [61].

### 5.2. Clinical Applications

The main difference between EIT and other lung imaging techniques lies in its ability to study global and regional lung ventilation dynamically and in real time. Most frequently, EIT has been used to monitor lung ventilation changes during ventilatory setting titration at the bedside [62,63,64]. Tidal impedance variation monitoring could potentially detect regional hyperinflation and dynamic overdistension, thus helping clinicians to set tidal volume; it can also help clinicians to identify pleural effusion, pneumothorax or endotracheal tube misplacement. The evaluation of regional distribution of ventilation, allowing for the detection of areas of hypoventilation and inhomogeneity, as well as EELI variation monitoring at different PEEP levels, makes EIT a valuable tool to titrate PEEP [65]. Indeed, EIT is able to assess both derecruitment and overinflation, theoretically leading to a reduction in ventilator-induced lung injury (VILI). Zhao et al. demonstrated that an EIT-based PEEP titration resulted in higher respiratory system compliance values (26 vs. 20 mL/cmH_2_O) as compared to a respiratory mechanics-based approach [66]; otherwise, Jimenez et al. demonstrated lower levels of mechanical power in the EIT-based PEEP titration group, along with a lower driving pressure and a higher respiratory system compliance [67]. The usefulness of EIT has been demonstrated to guide the application of adjunctive therapies, such as prone positioning [68], to evaluate the effect of recruitment maneuvers or PEEP titration in ameliorating dorsal ventilation and even in conditions of low tidal volume, as during veno-venous extracorporeal membrane oxygenation (ECMO). In addition, EIT has been used to assess the effects and effectiveness of routine maneuvers on the respiratory system, such as during tracheotomy [69], bronchoalveolar lavage [70], or endotracheal suctioning [71,72].

Observational studies have highlighted the EIT role during spontaneous breathing trial (SBT) and following extubation to assess lung aeration changes. Bosch-Compte et al. used EIT to compare different SBT methods with pressure support ventilation, demonstrating no difference in terms of aeration loss or respiratory effort [73]. Joussellin et al. found an association between the loss of lung volume assessed by EIT before extubation and the risk of extubation failure in mechanically ventilated patients with risk factors for extubation failure [74]. By the evaluation of tidal volume distribution, EIT suggested that an increase in the global inhomogeneity index could be a predictor of SBT failure [75,76].

**Figure 5:**
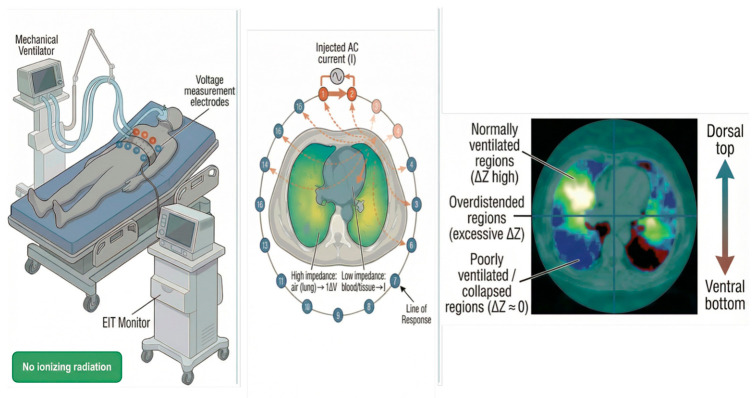
Instrumental setting (**Left**), electric emission and response acquisition pattern (**Center**), and image generation (**Right**) for electric impedance tomography (EIT). (**Left**): EIT can be used at the bedside as does not emit any ionizing radiation; (**Center**): electrodes are cyclically used as emitter/receiver of a low-voltage current to investigate local impedance variation in the thorax; (**Right**): EIT provides a functional dynamic image of a portion of the lung identifying regions with no, low, or high impedance variation from expiration to inspiration, according to their aeration.

Finally, EIT has been used to investigate lung perfusion in relation to ventilation distribution [59,77]; the amount of ventilation–perfusion mismatch during ARDS measured by EIT has been found to be associated with a greater mortality [78] (Figure 5).

### 5.3. Limitations

Its low spatial resolution precludes precise anatomical diagnosis, and because impedance measurements are inherently relative to a baseline, absolute values cannot be compared across patients. EIT is also blind to regions in which tidal impedance does not vary, such as atelectasis, pleural effusion, or large bullae, and end-expiratory lung volume cannot be directly quantified. Normal ranges for EIT-derived parameters remain undefined, and the clinical impact of EIT-guided ventilation strategies has yet to be robustly demonstrated [79].

## 6. Positron Emission Tomography

### 6.1. Physics and Overview of the Modality

Positron emission tomography (PET) is a functional imaging modality based on the detection of a radiotracer bound to a biological molecule, most commonly 2-deoxy-2-[^18^F]fluoro-D-glucose (^18^F-FDG). Positrons emitted by the tracer annihilate with local tissue electrons, generating pairs of photons that travel in opposite directions and are simultaneously captured by a ring-shaped detector; image reconstruction relies on the accurate measurement of their coincident arrival [14]. Several tracers have been investigated in experimental and clinical settings [80,81]. ^18^F-FDG, a glucose analog, enables the assessment of cellular glycolytic activity and is widely used in tissues with high glucose uptake, such as brain and tumors. Because activated neutrophils are also highly glycolytic, ^18^F-FDG-PET has proven useful for identifying foci of active infection in sepsis and for quantifying tissue inflammation [14]. In experimental studies of mechanical ventilation, [^13^N]-N_2_ has been used to quantify regional aeration and perfusion: inhaled [^13^N]-N_2_ washout correlates with regional lung aeration as measured by combined CT–PET, while intravenous administration (dissolved in saline) provides information on ventilation–perfusion mismatch [82,83,84,85].

### 6.2. Clinical Applications

As ARDS is characterized by an increased pulmonary vascular permeability, together with the coexistence of over-, normally, poorly and non-aerated lung regions, as demonstrated in detail by CT [19]; combining PET and CT scans with ^18^F-FDG enables the simultaneous assessment of regional lung aeration and inflammation distribution and magnitude within the lung. PET imaging has demonstrated that the lung metabolic activity is significantly higher in ARDS patients than in healthy subjects; however, it does not correlate with the relative weight of non-aerated or normally aerated tissues, but it is negatively associated with oxygenation levels [86]. Using a PET/CT approach, Bellani et al. demonstrated that a higher metabolic activity was present not only in non-aerated lung regions, but also in areas detected by CT as normally aerated [86]. This supports the concept that the baby lung is as affected by the inflammatory process as the rest of the lung, even though it maintains normal aeration. Indeed, the same authors recently demonstrated that lung regions undergoing intratidal recruitment and derecruitment exhibit similar inflammation with respect to a collapsed one [87].

**Figure 6:**
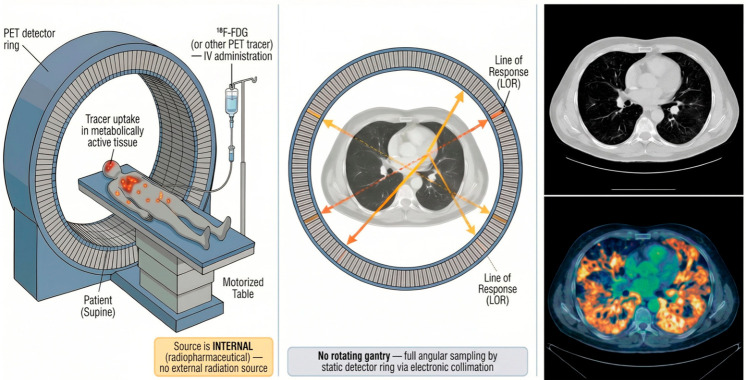
Instrumental setting (**Left**), response acquisition geometrical details (**Center**), and final rendering of computed tomography (CT) scan (**Upper Right**) and positron emission tomography (PET) scan (**Lower Right**). (**Left**): the intravenous contrast is metabolically active and is distributed into target tissues before the acquisition; therefore, the source of radiation is internal to the patient; (**Center**): the emission of divergent photons is sampled by a static detector ring. (**Lower Right**): PET image reconstruction with inflammation pattern, seen as different colors.

In patients with pneumonia, PET provides a quantitative assessment of pulmonary inflammation by measuring of uptake of radiotracers by activated inflammatory cells. The transcapillary escape rate has been shown to be significantly higher in areas that correspond to radiographic infiltrates [88] (Figure 6).

### 6.3. Limitations

The main limitations of using PET clinically are the cost and duration of the examination, as well as exposure to radiation and the difficulties and risks associated with transporting critically ill patients.

## 7. Future Directions

The landscape of lung imaging in critically ill patients is undergoing a profound transformation, driven by converging advances in artificial intelligence (AI), novel imaging physics, molecular biology, and miniaturized technology. Artificial intelligence is poised to fundamentally reshape diagnostic and therapeutic decision-making in respiratory critical care.

### 7.1. AI and CXR

Convoluted neural network-based algorithms have been compared to expert physicians on detecting ARDS from CXR, demonstrating a comparable performance with acceptable levels of sensitivity and specificity (83 and 88%, respectively) [89]. Broecker et al. demonstrated that a deep learning model integrated with a CXR combined with ventilator waveform data and clinical data acquired in patients admitted in the ICU within the first 24 h of intubation was able to improve ARDS classification [90].

### 7.2. AI and CT

CT is the most studied area of application of AI during AHRF in terms of automation of time-consuming tasks such as quantitative CT lung segmentation and the development of AI algorithms to predict the clinical trajectory of ARDS and stratify patients according to recruitability phenotype [91,92]. The integration of multimodal data streams, combining imaging features with physiological variables, blood biomarkers, and ventilator waveforms, will further enhance the predictive power of these models, potentially enabling lung recruitment prediction [93]. On the technological frontier, several emerging imaging modalities promise to overcome the intrinsic limitations of currently available techniques. While conventional quantitative CT characterizes lung parenchyma in terms of aeration and tissue density, dual-energy CT (DECT) simultaneously acquires image data at two different X-ray energy levels, enabling the decomposition of voxel content based on differential attenuation characteristics and allowing for the generation of iodine distribution maps from a single contrast-enhanced acquisition, essentially providing a surrogate measure of regional pulmonary blood volume across the entire lung [94]. DECT can map pulmonary blood volume throughout the whole lung with comparatively limited radiation exposure, making it feasible for application in clinical research settings. This technique could be useful in revealing a degree of vasculopathy that conventional CT morphology alone would have underestimated [17].

### 7.3. AI and LUS

After the COVID-19 pandemic, large datasets containing LUS images of patients with AHRF from different etiologies have been released to build machine learning- and deep learning-based algorithms used to automatically interpret LUS imaging and calculate standardized scores [38]. Although these algorithms exhibited a fair clinical accuracy in detecting COVID-19 pneumonia with respect to other etiologies, they can quantify the extent of lung damage and monitor the evolution of the disease [95,96,97,98,99].

### 7.4. AI and EIT

Recently, algorithms based on convolutional neural networks have been developed to improve EIT image quality and reconstruction and are publicly available (e.g., https://eidors3d.sourceforge.net/).

Moreover, machine learning has been used in some studies to model outcomes of patients with AHRF supported by high flow nasal cannulas and to predict post-extubation respiratory failure [100,101]. Moreover, advances in electrical impedance tomography will consolidate its transition from a research instrument to a routine clinical monitoring tool, enabling continuous, real-time visualization of regional ventilation distribution, dynamic strain, and PEEP-induced recruitment without radiation exposure or the need for patient transport.

### 7.5. AI and PET

Although PET remains a research tool in the area of AHRF, AI is entering PET largely through radiomics and combined PET/CT analysis. Nevertheless, most published works on AI-assisted PET still focus on oncology rather than ARDS specifically [88].

## 8. Conclusions

The available lung imaging techniques should be regarded as complementary, as no single imaging modality currently meets all the needs of critically ill patients with acute hypoxic respiratory failure (AHRF).

In the initial assessment of AHRF, lung ultrasound (LUS)—a radiation-free, bedside technique—has been shown to be highly accurate in diagnosing pneumothorax, pleural effusion, and interstitial syndromes, and can distinguish between cardiogenic and non-cardiogenic respiratory failure. In cases of non-cardiogenic respiratory failure, chest X-ray (CXR) retains a role as a rapid diagnostic tool, despite its limited sensitivity and substantial inter-observer variability. However, LUS is increasingly being incorporated into modern ARDS diagnostic criteria. In case of discrepancies between clinical severity and CXR findings, computed tomography (CT) remains the gold standard for confirming the diagnosis. Subsequently, in the first 48 h of AHRF, CT scans and electrical impedance tomography (EIT) can be useful for morphological and quantitative phenotyping, including the assessment of recruitability and the optimization of mechanical ventilation settings and response to the prone position. LUS can provide daily monitoring of the disease course and CT can set in in case of sudden clinical worsening. In the late stages of the disease, positron emission tomography (PET) could potentially provide insight into regional inflammation and the evolution of the fibroproliferative phase, although it is currently only used in research. Therefore, the rational, modality-specific use of these techniques, matched to the clinical question and the time course of the disease, could enable a more personalized approach to AHRF. The recent converging innovations point to a future in which lung imaging moves beyond its traditional role as a passive diagnostic modality to become an active, continuous, and personalized guide to respiratory management. The future prospect of AI-driven, closed-loop ventilation systems—capable of autonomously integrating real-time imaging with physiological monitoring to deliver adaptive, injury-minimizing ventilatory support—could have the potential to reshape the interplay between imaging, monitoring, and therapy in respiratory critical care.

## Figures and Tables

**Table 1:** Summary of the main clinical indications, and the pros and cons of each lung imaging technique. PTX: pneumothorax; PEEP: positive end-expiratory pressure; Va/Q; ventilation–perfusion; ET: endotracheal tube.

Technique	Indications	Pros	Cons
Chest X-Ray (CXR)	Most frequently used tool for initial assessment Checking correct placement of devices Detection of sudden clinical worsening	Repeatable and bedside Low doses of ionizing radiation	Low sensitivity Significant inter-observer variability
Computed Tomography (CT)	Identification of morphological phenotypes Identification of pleural effusions Identification of fibroproliferative processes Identification of complications during mechanical ventilation (PTX, pneumomediastinum)	High clinical accuracy Pulmonary embolism identification Precise quantification of regional aeration Assessment of lung recruitability and overdistension	Need for critically ill patient transport Exposure to a cumulatively high level of ionizing radiation
Lung Ultrasound (LUS)	Assessment of cardiogenic interstitial syndrome Daily monitoring of lung re-aeration Bedside diagnosis of suspected PTX Quantification of pleural effusion Quantification of response to prone positioning and to PEEP	Repeatable and bedside Assessment of focal vs. non-focal morphology Availability of semi-quantitative scores High diagnostic accuracy for PTX High diagnostic accuracy for pleural effusion No exposure to ionizing radiation	Insensitive to over-distension Significant inter-observer variability Based on indirect artifacts analysis
Electrical Impedance Tomography (EIT)	PEEP titration during mechanical ventilation Detection of collapse, overdistension, and *pendelluft* during mechanical ventilation Quantification of response to prone positioning Bedside Va/Q mismatch analysis	Repeatable and bedside Real-time global and regional aeration analysis during both spontaneous breathing and mechanical ventilation Detection of ET misplacement and PTX Possibility to assess lung perfusion No exposure to ionizing radiation	Poor availability Spatially limited analysis to belt positioning Unable to provide anatomical diagnosis Unable to identify non-aerated areas (atelectasis, pleural effusion, large *bullae*) Absence of normal ranges of EIT-derived parameters
Positron Emission Tomography (PET)	Not routinely used in clinical practice Mapping inflammatory activity Va/Q mismatch and pulmonary vascular permeability analysis	Quantitative assessment of lung inflammation Quantitative assessment of both ventilation and perfusion	Need for a radioactive tracer Need for critically ill patient transport Exposure to a cumulatively high level of ionizing radiation

**Table 2:** Average ionizing radiation exposure according to the type of computed tomography (CT). CXR: chest X-ray.

Type of CT	Average Dose	CXR Equivalents	Notes
Low-dose CT	1.5 mSv	~25–75	Used for screening
High-resolution CT	1 mSv	~100–200	Used for fibrosis and interstitial lung diseases
Standard CT	7 mSv	~200–350	Most common in the emergency department
CT pulmonary angiogram	15 mSv	~400–750	High resolution and fast acquisition

## Data Availability

No new data were created or analyzed in this study.

## Abbreviations

The following abbreviations are used in this manuscript:

AHRF	Acute hypoxemic respiratory failure
ARDS	Acute respiratory distress syndrome
CXR	Chest X-ray
CT	Computed tomography
LUS	Lung ultrasound
CPIS	Clinical Pulmonary Infection Score
EIT	Electric impedance tomography
TIV	Tidal impedance variation
ROIs	Regions of interest
PEEP	Positive end-expiratory pressure
VILI	Ventilator-induced lung injury
ECMO	Extracorporeal membrane oxygenation
SBT	Spontaneous breathing trial
PET	Positron emission tomography
^18^F-FDG	[^18^F]-fluoro-2-deoxy-D-glucose
AI	Artificial intelligence
DECT	Dual-energy computed tomography

## References

[1] Perez J., Brandan L., Telias I. (2025). Monitoring Patients with Acute Respiratory Failure during Non-Invasive Respiratory Support to Minimize Harm and Identify Treatment Failure. Crit. Care.

[2] Lagina M., Valley T.S. (2024). Diagnosis and Management of Acute Respiratory Failure. Crit. Care Clin..

[3] Chiumello D., Sferrazza Papa G.F., Artigas A., Bouhemad B., Grgic A., Heunks L., Markstaller K., Pellegrino G.M., Pisani L., Rigau D. (2019). ERS Statement on Chest Imaging in Acute Respiratory Failure. Eur. Respir. J..

[4] Schalekamp S., van Ginneken B., Karssemeijer N., Schaefer-Prokop C.M. (2014). Chest Radiography: New Technological Developments and Their Applications. Semin. Respir. Crit. Care Med..

[5] Eisenhuber E., Schaefer-Prokop C.M., Prosch H., Schima W. (2012). Bedside Chest Radiography. Respir. Care.

[6] Bentz M.R., Primack S.L. (2015). Intensive Care Unit Imaging. Clin. Chest Med..

[7] Wassipaul C., Janata-Schwatczek K., Domanovits H., Tamandl D., Prosch H., Scharitzer M., Polanec S., Schernthaner R.E., Mang T., Asenbaum U. (2023). Ultra-Low-Dose CT vs. Chest X-Ray in Non-Traumatic Emergency Department Patients—A Prospective Randomised Crossover Cohort Trial. EClinicalMedicine.

[8] Oba Y., Zaza T. (2010). Abandoning Daily Routine Chest Radiography in the Intensive Care Unit: Meta-Analysis. Radiology.

[9] Ganapathy A., Adhikari N.K., Spiegelman J., Scales D.C. (2012). Routine Chest X-Rays in Intensive Care Units: A Systematic Review and Meta-Analysis. Crit. Care.

[10] Winkler M.H., Touw H.R., van de Ven P.M., Twisk J., Tuinman P.R. (2018). Diagnostic Accuracy of Chest Radiograph, and When Concomitantly Studied Lung Ultrasound, in Critically Ill Patients with Respiratory Symptoms: A Systematic Review and Meta-Analysis. Crit. Care Med..

[11] Brooks D., Wright S.E., Beattie A., McAllister N., Anderson N.H., Roy A.I., Gonsalves P., Yates B., Graziadio S., Mackie A. (2024). Assessment of the Comparative Agreement between Chest Radiographs and CT Scans in Intensive Care Units. J. Crit. Care.

[12] Larke F.J., Kruger R.L., Cagnon C.H., Flynn M.J., McNitt-Gray M.M., Wu X., Judy P.F., Cody D.D. (2011). Estimated Radiation Dose Associated with Low-Dose Chest CT of Average-Size Participants in the National Lung Screening Trial. Am. J. Roentgenol..

[13] Van der Bruggen-Bogaarts B.A.H.A., Broerse J.J., Lammers J.-W.J., Van Waes P.F.G.M., Geleijns J. (1995). Radiation Exposure in Standard and High-Resolution Chest CT Scans. Chest.

[14] Cereda M., Xin Y., Goffi A., Herrmann J., Kaczka D.W., Kavanagh B.P., Perchiazzi G., Yoshida T., Rizi R.R. (2019). Imaging the Injured Lung. Anesthesiology.

[15] (2012). The ARDS Definition Task Force. Acute Respiratory Distress Syndrome: The Berlin Definition. JAMA.

[16] Matthay M.A., Arabi Y., Arroliga A.C., Bernard G., Bersten A.D., Brochard L.J., Calfee C.S., Combes A., Daniel B.M., Ferguson N.D. (2024). A New Global Definition of Acute Respiratory Distress Syndrome. Am. J. Respir. Crit. Care Med..

[17] Chiumello D., Busana M., Coppola S., Romitti F., Formenti P., Bonifazi M., Pozzi T., Palumbo M.M., Cressoni M., Herrmann P. (2020). Physiological and Quantitative CT-Scan Characterization of COVID-19 and Typical ARDS: A Matched Cohort Study. Intensive Care Med..

[18] Constantin J.-M., Jabaudon M., Lefrant J.-Y., Jaber S., Quenot J.-P., Langeron O., Ferrandière M., Grelon F., Seguin P., Ichai C. (2019). Personalised Mechanical Ventilation Tailored to Lung Morphology versus Low Positive End-Expiratory Pressure for Patients with Acute Respiratory Distress Syndrome in France (the LIVE Study): A Multicentre, Single-Blind, Randomised Controlled Trial. Lancet Respir. Med..

[19] Gattinoni L., Caironi P., Pelosi P., Goodman L.R. (2001). What Has Computed Tomography Taught Us about the Acute Respiratory Distress Syndrome?. Am. J. Respir. Crit. Care Med..

[20] Gattinoni L., Mascheroni D., Torresin A., Marcolin R., Fumagalli R., Vesconi S., Rossi G.P., Rossi F., Baglioni S., Bassi F. (1986). Morphological Response to Positive End Expiratory Pressure in Acute Respiratory Failure. Computerized Tomography Study. Intensive Care Med..

[21] Pelosi P., D’Andrea L., Vitale G., Pesenti A., Gattinoni L. (1994). Vertical Gradient of Regional Lung Inflation in Adult Respiratory Distress Syndrome. Am. J. Respir. Crit. Care Med..

[22] (2000). Acute Respiratory Distress Syndrome Network. Ventilation with Lower Tidal Volumes as Compared with Traditional Tidal Volumes for Acute Lung Injury and the Acute Respiratory Distress Syndrome. N. Engl. J. Med..

[23] Gattinoni L., Caironi P., Cressoni M., Chiumello D., Ranieri V.M., Quintel M., Russo S., Patroniti N., Cornejo R., Bugedo G. (2006). Lung Recruitment in Patients with the Acute Respiratory Distress Syndrome. N. Engl. J. Med..

[24] Borges J.B., Carvalho C.R.R., Amato M.B.P. (2006). Lung Recruitment in Patients with ARDS. N. Engl. J. Med..

[25] Borges J.B., Okamoto V.N., Matos G.F.J., Caramez M.P.R., Arantes P.R., Barros F., Souza C.E., Victorino J.A., Kacmarek R.M., Barbas C.S.V. (2006). Reversibility of Lung Collapse and Hypoxemia in Early Acute Respiratory Distress Syndrome. Am. J. Respir. Crit. Care Med..

[26] Malbouisson L.M., Muller J.-C., Constantin J.-M., Lu Q., Puybasset L., Rouby J.-J. (2001). Computed Tomography Assessment of Positive End-Expiratory Pressure-Induced Alveolar Recruitment in Patients with Acute Respiratory Distress Syndrome. Am. J. Respir. Crit. Care Med..

[27] Gattinoni L., Pelosi P., Vitale G., Pesenti A., D’Andrea L., Mascheroni D. (1991). Body Position Changes Redistribute Lung Computed-Tomographic Density in Patients with Acute Respiratory Failure. Anesthesiology.

[28] Cornejo R.A., Díaz J.C., Tobar E.A., Bruhn A.R., Ramos C.A., González R.A., Repetto C.A., Romero C.M., Gálvez L.R., Llanos O. (2013). Effects of Prone Positioning on Lung Protection in Patients with Acute Respiratory Distress Syndrome. Am. J. Respir. Crit. Care Med..

[29] Guérin C., Reignier J., Richard J.-C., Beuret P., Gacouin A., Boulain T., Mercier E., Badet M., Mercat A., Baudin O. (2013). Prone Positioning in Severe Acute Respiratory Distress Syndrome. N. Engl. J. Med..

[30] Yang S., Sun Q., Yuan X., Wang J., Wang H., Hu W., Peng Q., Zhang C., Li X., Huang W. (2025). Effect of Prone Position on Ventilation-Perfusion Matching in Patients with Moderate to Severe ARDS with Different Clinical Phenotypes. Respir. Res..

[31] Knight P.H., Maheshwari N., Hussain J., Scholl M., Hughes M., Papadimos T.J., Guo W.A., Cipolla J., Stawicki S.P., Latchana N. (2015). Complications during Intrahospital Transport of Critically Ill Patients: Focus on Risk Identification and Prevention. Int. J. Crit. Illn. Inj. Sci..

[32] Formenti P., Carlucci P., Radovanovic D., Bruno G., Soldati G., Tursi F. (2025). Lung Re-Aeration Assessment by Ultrasound during Mechanical Ventilation: Current Knowledge of Literature Review. Multidiscip. Respir. Med..

[33] Volpicelli G., Elbarbary M., Blaivas M., Lichtenstein D.A., Mathis G., Kirkpatrick A.W., Melniker L., Gargani L., Noble V.E., Via G. (2012). International Evidence-Based Recommendations for Point-of-Care Lung Ultrasound. Intensive Care Med..

[34] Battaglini D., Schultz M.J., Puentes G.A.C., Marini J.J., Rocco P.R.M. (2025). Imaging and Pulmonary Function Techniques in ARDS Diagnosis and Management: Current Insights and Challenges. Crit. Care.

[35] Boumans M.M.A., Aerts W., Pisani L., Bos L.D.J., Smit M.R., Tuinman P.R. (2024). Diagnostic Accuracy of Lung Ultrasound in Diagnosis of ARDS and Identification of Focal or Non-Focal ARDS Subphenotypes: A Systematic Review and Meta-Analysis. Crit. Care.

[36] Song I.-K., Kim E.-H., Lee J.-H., Ro S., Kim H.-S., Kim J.-T. (2017). Effects of an Alveolar Recruitment Manoeuvre Guided by Lung Ultrasound on Anaesthesia-induced Atelectasis in Infants: A Randomised, Controlled Trial. Anaesthesia.

[37] Mongodi S., Pozzi M., Orlando A., Bouhemad B., Stella A., Tavazzi G., Via G., Iotti G.A., Mojoli F. (2018). Lung Ultrasound for Daily Monitoring of ARDS Patients on Extracorporeal Membrane Oxygenation: Preliminary Experience. Intensive Care Med..

[38] Wang J., Yang X., Zhou B., Sohn J.J., Zhou J., Jacob J.T., Higgins K.A., Bradley J.D., Liu T. (2022). Review of Machine Learning in Lung Ultrasound in COVID-19 Pandemic. J. Imaging.

[39] Prat G., Guinard S., Bizien N., Nowak E., Tonnelier J.-M., Alavi Z., Renault A., Boles J.-M., L’Her E. (2016). Can Lung Ultrasonography Predict Prone Positioning Response in Acute Respiratory Distress Syndrome Patients?. J. Crit. Care.

[40] Pichette M., Goffi A. (2018). A 45-Year-Old Man with Severe Respiratory Failure After Cardiac Arrest. Chest.

[41] Haddam M., Zieleskiewicz L., Perbet S., Baldovini A., Guervilly C., Arbelot C., Noel A., Vigne C., Hammad E., Antonini F. (2016). Lung Ultrasonography for Assessment of Oxygenation Response to Prone Position Ventilation in ARDS. Intensive Care Med..

[42] Bouhemad B., Brisson H., Le-Guen M., Arbelot C., Lu Q., Rouby J.-J. (2011). Bedside Ultrasound Assessment of Positive End-Expiratory Pressure–Induced Lung Recruitment. Am. J. Respir. Crit. Care Med..

[43] Soldati G., Inchingolo R., Smargiassi A., Sher S., Nenna R., Inchingolo C.D., Valente S. (2012). Ex Vivo Lung Sonography: Morphologic-Ultrasound Relationship. Ultrasound Med. Biol..

[44] Enghard P., Rademacher S., Nee J., Hasper D., Engert U., Jörres A., Kruse J.M. (2015). Simplified Lung Ultrasound Protocol Shows Excellent Prediction of Extravascular Lung Water in Ventilated Intensive Care Patients. Crit. Care.

[45] Volpicelli G., Skurzak S., Boero E., Carpinteri G., Tengattini M., Stefanone V., Luberto L., Anile A., Cerutti E., Radeschi G. (2014). Lung Ultrasound Predicts Well Extravascular Lung Water but Is of Limited Usefulness in the Prediction of Wedge Pressure. Anesthesiology.

[46] Lichtenstein D.A., Mezière G.A., Lagoueyte J.-F., Biderman P., Goldstein I., Gepner A. (2009). A-Lines and B-Lines. Chest.

[47] Tsuchida S., Engelberts D., Peltekova V., Hopkins N., Frndova H., Babyn P., McKerlie C., Post M., McLoughlin P., Kavanagh B.P. (2006). Atelectasis Causes Alveolar Injury in Nonatelectatic Lung Regions. Am. J. Respir. Crit. Care Med..

[48] Alrajab S., Youssef A.M., Akkus N.I., Caldito G. (2013). Pleural Ultrasonography versus Chest Radiography for the Diagnosis of Pneumothorax: Review of the Literature and Meta-Analysis. Crit. Care.

[49] Dulchavsky S.A., Schwarz K.L., Kirkpatrick A.W., Billica R.D., Williams D.R., Diebel L.N., Campbell M.R., Sargysan A.E., Hamilton D.R. (2001). Prospective Evaluation of Thoracic Ultrasound in the Detection of Pneumothorax. J. Trauma Inj. Infect. Crit. Care.

[50] Kirkpatrick A.W., Sirois M., Laupland K.B., Liu D., Rowan K., Ball C.G., Hameed S.M., Brown R., Simons R., Dulchavsky S.A. (2004). Hand-Held Thoracic Sonography for Detecting Post-Traumatic Pneumothoraces: The Extended Focused Assessment with Sonography for Trauma (EFAST). J. Trauma Inj. Infect. Crit. Care.

[51] Husain L., Hagopian L., Wayman D., Baker W., Carmody K. (2012). Sonographic Diagnosis of Pneumothorax. J. Emerg. Trauma Shock.

[52] Vignon P., Chastagner C., Berkane V., Chardac E., François B., Normand S., Bonnivard M., Clavel M., Pichon N., Preux P.-M. (2005). Quantitative Assessment of Pleural Effusion in Critically Ill Patients by Means of Ultrasonography. Crit. Care Med..

[53] Mayo P.H., Goltz H.R., Tafreshi M., Doelken P. (2004). Safety of Ultrasound-Guided Thoracentesis in Patients Receiving Mechanical Ventilation. Chest.

[54] Mongodi S., Via G., Girard M., Rouquette I., Misset B., Braschi A., Mojoli F., Bouhemad B. (2016). Lung Ultrasound for Early Diagnosis of Ventilator-Associated Pneumonia. Chest.

[55] Lichtenstein D., Mezière G., Seitz J. (2009). The Dynamic Air Bronchogram. Chest.

[56] Xuan N., Tian B., Ying L., Cao X., Wang D., Zhang G. (2025). Electrical Impedance Tomography: From Technical Innovations to Bedside Clinical Solutions. World J. Emerg. Med..

[57] Spinelli E., Mauri T., Fogagnolo A., Scaramuzzo G., Rundo A., Grieco D.L., Grasselli G., Volta C.A., Spadaro S. (2019). Electrical Impedance Tomography in Perioperative Medicine: Careful Respiratory Monitoring for Tailored Interventions. BMC Anesthesiol..

[58] Rauseo M., Spinelli E., Sella N., Slobod D., Spadaro S., Longhini F., Giarratano A., Gilda C., Mauri T., Navalesi P. (2022). Expert Opinion Document: “Electrical Impedance Tomography: Applications from the Intensive Care Unit and Beyond”. J. Anesth. Analg. Crit. Care.

[59] Leali M., Fujitani S., Mauri T. (2026). Ventilation/Perfusion Mismatch Measured by Electrical Impedance Tomography at the Bedside: Potentialities and Challenges. Intensive Care Med..

[60] Borges J.B., Suarez-Sipmann F., Bohm S.H., Tusman G., Melo A., Maripuu E., Sandström M., Park M., Costa E.L.V., Hedenstierna G. (2012). Regional Lung Perfusion Estimated by Electrical Impedance Tomography in a Piglet Model of Lung Collapse. J. Appl. Physiol..

[61] Scaramuzzo G., Pavlovsky B., Adler A., Baccinelli W., Bodor D.L., Damiani L.F., Franchineau G., Francovich J., Frerichs I., Giralt J.A.S. (2024). Electrical Impedance Tomography Monitoring in Adult ICU Patients: State-of-the-Art, Recommendations for Standardized Acquisition, Processing, and Clinical Use, and Future Directions. Crit. Care.

[62] Pulletz S., Kott M., Elke G., Schädler D., Vogt B., Weiler N., Frerichs I. (2012). Dynamics of Regional Lung Aeration Determined by Electrical Impedance Tomography in Patients with Acute Respiratory Distress Syndrome. Multidiscip. Respir. Med..

[63] Cinnella G., Grasso S., Raimondo P., D’Antini D., Mirabella L., Rauseo M., Dambrosio M. (2015). Physiological Effects of the Open Lung Approach in Patients with Early, Mild, Diffuse Acute Respiratory Distress Syndrome. Anesthesiology.

[64] Luecke T., Corradi F., Pelosi P. (2012). Lung Imaging for Titration of Mechanical Ventilation. Curr. Opin. Anaesthesiol..

[65] Yuan X., Zhong M., Li Z., Sang L., Huang X., Zhang R., Chen H., Gao Y., Wang Y., Lin Z. (2026). Electrical Impedance Tomography-Guided Positive end-Expiratory Pressure and Mortality of Patients with the Acute Respiratory Distress Syndrome the EITVent Randomized Controlled Trial 2025. Am. J. Respir. Crit. Care Med..

[66] Zhao Z., Chang M.-Y., Chang M.-Y., Gow C.-H., Zhang J.-H., Hsu Y.-L., Frerichs I., Chang H.-T., Möller K. (2019). Positive End-Expiratory Pressure Titration with Electrical Impedance Tomography and Pressure–Volume Curve in Severe Acute Respiratory Distress Syndrome. Ann. Intensive Care.

[67] Jimenez J.V., Munroe E., Weirauch A.J., Fiorino K., Culter C.A., Nelson K., Labaki W.W., Choi P.J., Co I., Standiford T.J. (2023). Electric Impedance Tomography-Guided PEEP Titration Reduces Mechanical Power in ARDS: A Randomized Crossover Pilot Trial. Crit. Care.

[68] Pierrakos C., van der Ven F.L.I.M., Smit M.R., Hagens L.A., Paulus F., Schultz M.J., Bos L.D.J. (2022). Prone Positioning Decreases Inhomogeneity and Improves Dorsal Compliance in Invasively Ventilated Spontaneously Breathing COVID-19 Patients—A Study Using Electrical Impedance Tomography. Diagnostics.

[69] Eichler L., Mueller J., Grensemann J., Frerichs I., Zöllner C., Kluge S. (2018). Lung Aeration and Ventilation after Percutaneous Tracheotomy Measured by Electrical Impedance Tomography in Non-Hypoxemic Critically Ill Patients: A Prospective Observational Study. Ann. Intensive Care.

[70] Frerichs I., Dargaville P.A., Rimensberger P.C. (2019). Regional Pulmonary Effects of Bronchoalveolar Lavage Procedure Determined by Electrical Impedance Tomography. Intensive Care Med. Exp..

[71] Lindgren S., Odenstedt H., Olegård C., Söndergaard S., Lundin S., Stenqvist O. (2007). Regional Lung Derecruitment after Endotracheal Suction during Volume- or Pressure-Controlled Ventilation: A Study Using Electric Impedance Tomography. Intensive Care Med..

[72] Chiumello D., Bolgiaghi L., Formenti P., Pozzi T., Lucenteforte M., Coppola S. (2021). Effects on Lung Gas Volume, Respiratory Mechanics and Gas Exchange of a Closed-Circuit Suctioning System during Volume- and Pressure-Controlled Ventilation in ARDS Patients. J. Clin. Med..

[73] Bosch-Compte R., Parrilla F.J., Muñoz-Bermúdez R., Dot I., Climent C., Masclans J.R., Marin-Corral J., Pérez-Terán P. (2024). Comparing Lung Aeration and Respiratory Effort Using Two Different Spontaneous Breathing Trial: T-Piece vs Pressure Support Ventilation. Med. Intensiv..

[74] Joussellin V., Bonny V., Spadaro S., Clerc S., Parfait M., Ferioli M., Sieye A., Jalil Y., Janiak V., Pinna A. (2023). Lung Aeration Estimated by Chest Electrical Impedance Tomography and Lung Ultrasound during Extubation. Ann. Intensive Care.

[75] Coppadoro A., Grassi A., Giovannoni C., Rabboni F., Eronia N., Bronco A., Foti G., Fumagalli R., Bellani G. (2020). Occurrence of Pendelluft under Pressure Support Ventilation in Patients Who Failed a Spontaneous Breathing Trial: An Observational Study. Ann. Intensive Care.

[76] Longhini F., Maugeri J., Andreoni C., Ronco C., Bruni A., Garofalo E., Pelaia C., Cavicchi C., Pintaudi S., Navalesi P. (2019). Electrical Impedance Tomography during Spontaneous Breathing Trials and after Extubation in Critically Ill Patients at High Risk for Extubation Failure: A Multicenter Observational Study. Ann. Intensive Care.

[77] Takai D., Maeda A., Yamamoto M., Fujishiro K., Hattammaru Y., Inokuchi R., Kawashima M., Konoeda C., Sato M., Doi K. (2026). Visualizing and Quantifying Ventilation-Perfusion Mismatch by Electrical Impedance Tomography in Patients Undergoing Lung Transplantation: A Pilot Physiological Study. Transplant. Proc..

[78] Spinelli E., Kircher M., Stender B., Ottaviani I., Basile M.C., Marongiu I., Colussi G., Grasselli G., Pesenti A., Mauri T. (2021). Unmatched Ventilation and Perfusion Measured by Electrical Impedance Tomography Predicts the Outcome of ARDS. Crit. Care.

[79] Costa E.L., Lima R.G., Amato M.B. (2009). Electrical Impedance Tomography. Curr. Opin. Crit. Care.

[80] Dimastromatteo J., Charles E.J., Laubach V.E. (2018). Molecular Imaging of Pulmonary Diseases. Respir. Res..

[81] Chen D.L., Schiebler M.L., Goo J.M., van Beek E.J.R. (2017). PET Imaging Approaches for Inflammatory Lung Diseases: Current Concepts and Future Directions. Eur. J. Radiol..

[82] Simon B.A., Venegas J.G. (1994). Analyzing 13NN Lung Washout Curves in the Presence of Intraregional Nonuniformities. J. Appl. Physiol..

[83] Galletti G.G., Venegas J.G. (2002). Tracer Kinetic Model of Regional Pulmonary Function Using Positron Emission Tomography. J. Appl. Physiol..

[84] O’Neill K., Venegas J.G., Richter T., Harris R.S., Layfield J.D.H., Musch G., Winkler T., Melo M.F.V. (2003). Modeling Kinetics of Infused 13NN-Saline in Acute Lung Injury. J. Appl. Physiol..

[85] Musch G., Harris R.S., Vidal Melo M.F., O’Neill K.R., Layfield J.D.H., Winkler T., Venegas J.G. (2004). Mechanism by Which a Sustained Inflation Can Worsen Oxygenation in Acute Lung Injury. Anesthesiology.

[86] Bellani G., Messa C., Guerra L., Spagnolli E., Foti G., Patroniti N., Fumagalli R., Musch G., Fazio F., Pesenti A. (2009). Lungs of Patients with Acute Respiratory Distress Syndrome Show Diffuse Inflammation in Normally Aerated Regions: A [18F]-Fluoro-2-Deoxy-D-Glucose PET/CT Study. Crit. Care Med..

[87] Bellani G., Rouby J.-J., Constantin J.-M., Pesenti A. (2017). Looking Closer at Acute Respiratory Distress Syndrome: The Role of Advanced Imaging Techniques. Curr. Opin. Crit. Care.

[88] Bellani G., Mauri T., Pesenti A. (2012). Imaging in Acute Lung Injury and Acute Respiratory Distress Syndrome. Curr. Opin. Crit. Care.

[89] Sjoding M.W., Taylor D., Motyka J., Lee E., Co I., Claar D., McSparron J.I., Ansari S., Kerlin M.P., Reilly J.P. (2021). Deep Learning to Detect Acute Respiratory Distress Syndrome on Chest Radiographs: A Retrospective Study with External Validation. Lancet Digit. Health.

[90] Broecker S., Adams J.Y., Kumar G., Callcut R.A., Ni Y., Strohmer T. (2025). Multimodal Deep Learning for ARDS Detection 2025. medRxiv.

[91] Herrmann P., Busana M., Cressoni M., Lotz J., Moerer O., Saager L., Meissner K., Quintel M., Gattinoni L. (2021). Using Artificial Intelligence for Automatic Segmentation of CT Lung Images in Acute Respiratory Distress Syndrome. Front. Physiol..

[92] Xin Y., Song G., Cereda M., Kadlecek S., Hamedani H., Jiang Y., Rajaei J., Clapp J., Profka H., Meeder N. (2015). Semiautomatic Segmentation of Longitudinal Computed Tomography Images in a Rat Model of Lung Injury by Surfactant Depletion. J. Appl. Physiol..

[93] Pennati F., Aliverti A., Pozzi T., Gattarello S., Lombardo F., Coppola S., Chiumello D. (2023). Machine Learning Predicts Lung Recruitment in Acute Respiratory Distress Syndrome Using Single Lung CT Scan. Ann. Intensive Care.

[94] Gaulton T.G., Xin Y., Victor M., Nova A., Cereda M. (2024). Imaging the Pulmonary Vasculature in Acute Respiratory Distress Syndrome. Nitric Oxide.

[95] Roy S., Menapace W., Oei S., Luijten B., Fini E., Saltori C., Huijben I., Chennakeshava N., Mento F., Sentelli A. (2020). Deep Learning for Classification and Localization of COVID-19 Markers in Point-of-Care Lung Ultrasound. IEEE Trans. Med. Imaging.

[96] Mento F., Perrone T., Fiengo A., Smargiassi A., Inchingolo R., Soldati G., Demi L. (2021). Deep Learning Applied to Lung Ultrasound Videos for Scoring COVID-19 Patients: A Multicenter Study. J. Acoust. Soc. Am..

[97] Demi L., Mento F., Di Sabatino A., Fiengo A., Sabatini U., Macioce V.N., Robol M., Tursi F., Sofia C., Di Cienzo C. (2022). Lung Ultrasound in COVID-19 and Post-COVID-19 Patients, an Evidence-Based Approach. J. Ultrasound Med..

[98] Erfanian Ebadi S., Krishnaswamy D., Bolouri S.E.S., Zonoobi D., Greiner R., Meuser-Herr N., Jaremko J.L., Kapur J., Noga M., Punithakumar K. (2021). Automated Detection of Pneumonia in Lung Ultrasound Using Deep Video Classification for COVID-19. Inform. Med. Unlocked.

[99] La Salvia M., Secco G., Torti E., Florimbi G., Guido L., Lago P., Salinaro F., Perlini S., Leporati F. (2021). Deep Learning and Lung Ultrasound for COVID-19 Pneumonia Detection and Severity Classification. Comput. Biol. Med..

[100] Yang L., Li Z., Dai M., Fu F., Möller K., Gao Y., Zhao Z. (2023). Optimal Machine Learning Methods for Prediction of High-Flow Nasal Cannula Outcomes Using Image Features from Electrical Impedance Tomography. Comput. Methods Programs Biomed..

[101] Rubin J., Berra L. (2022). Electrical Impedance Tomography in the Adult Intensive Care Unit: Clinical Applications and Future Directions. Curr. Opin. Crit. Care.

